# Supplementary material for: Causal Relationship between Aging and Anorexia Nervosa: A White-Matter-Microstructure-Mediated Mendelian Randomization Analysis
Source: Biomedicines. 2024 Aug 16;12(8):1874. doi: 10.3390/biomedicines12081874 (PMC11351342; doi:10.3390/biomedicines12081874)
Supplement: Supplementary file 1 [file biomedicines-12-01874-s001.zip › Supplementary Figure/Figure S4/Singe SNP plot of MR analysis for TL to ubm-b-2061.pdf]

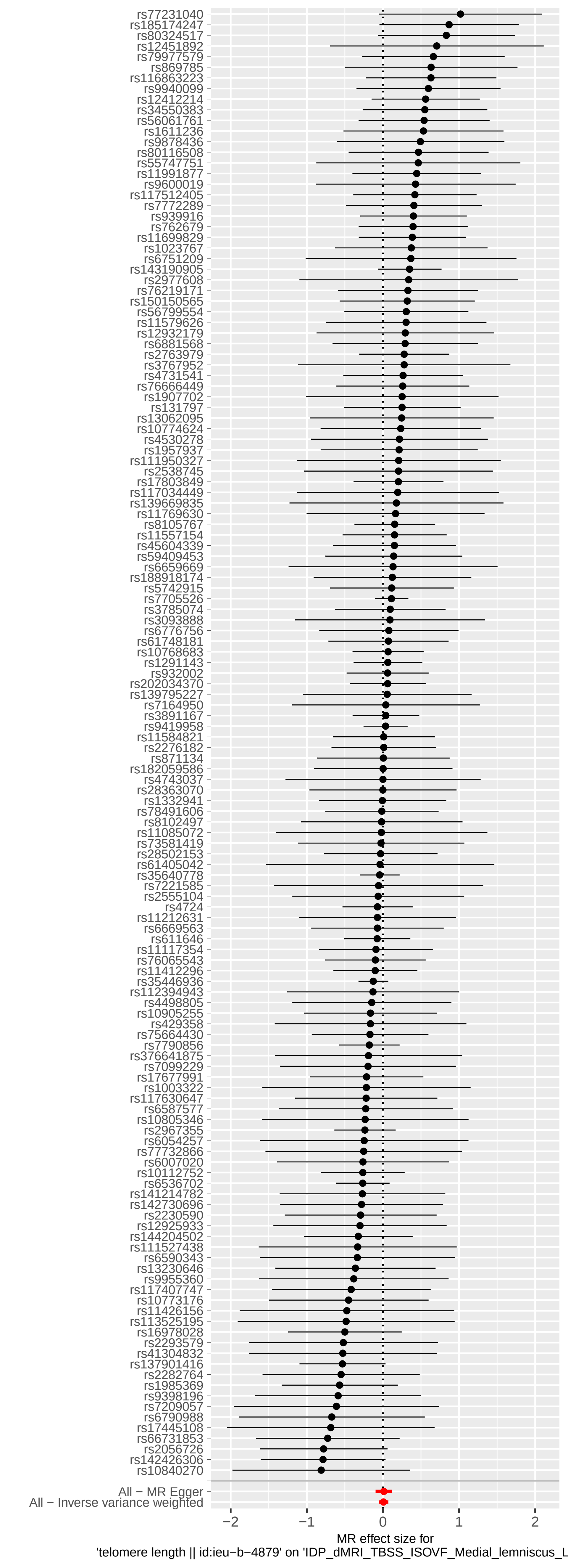
rs77231040
rs185174247
rs80324517
rs12451892
rs79977579
rs869785
rs116863223
rs9940099
rs12412214
rs34550383
rs56061761
rs1611236
rs9878436
rs80116508
rs55747751
rs11991877
rs9600019
rs117512405
rs7772289
rs939916
rs762679
rs11699829
rs1023767
rs6751209
rs143190905
rs2977608
rs76219171
rs150150565
rs56799554
rs11579626
rs12932179
rs6881568
rs2763979
rs3767952
rs4731541
rs76666449
rs1907702
rs131797
rs13062095
rs10774624
rs4530278
rs1957937
rs111950327
rs2538745
rs17803849
rs117034449
rs139669835
rs11769630
rs8105767
rs11557154
rs45604339
rs59409453
rs6659669
rs188918174
rs5742915
rs7705526
rs3785074
rs3093888
rs6776756
rs61748181
rs10768683
rs1291143
rs932002
rs202034370
rs139795227
rs7164950
rs3891167
rs9419958
rs11584821
rs2276182
rs871134
rs182059586
rs4743037
rs28363070
rs1332941
rs78491606
rs8102497
rs11085072
rs73581419
rs28502153
rs61405042
rs35640778
rs7221585
rs2555104
rs4724
rs11212631
rs6669563
rs611646
rs11117354
rs76065543
rs11412296
rs35446936
rs112394943
rs4498805
rs10905255
rs429358
rs75664430
rs7790856
rs376641875
rs7099229
rs17677991
rs1003322
rs117630647
rs6587577
rs10805346
rs2967355
rs6054257
rs77732866
rs6007020
rs10112752
rs6536702
rs141214782
rs142730696
rs2230590
rs12925933
rs144204502
rs111527438
rs6590343
rs13230646
rs9955360
rs117407747
rs10773176
rs11426156
rs113525195
rs16978028
rs2293579
rs41304832
rs137901416
rs2282764
rs1985369
rs9398196
rs7209057
rs6790988
rs17445108
rs66731853
rs2056726
rs142426306
rs10840270
All − MR Egger
All − Inverse variance weighted
−2
−1
0
1
2
MR effect size for
'telomere length || id:ieu−b−4879' on 'IDP_dMRI_TBSS_ISOVF_Medial_lemniscus_L
